# Outlook of Cassava Brown Streak Disease Assessment: Perspectives of the Screening Methods of Breeders and Pathologists

**DOI:** 10.3389/fpls.2021.648436

**Published:** 2021-07-05

**Authors:** Alfred A. Ozimati, Williams Esuma, Titus Alicai, Jean-Luc Jannink, Chiedozie Egesi, Robert Kawuki

**Affiliations:** ^1^Root Crops Program, National Crops Resources Research Institute (NaCRRI), Kampala, Uganda; ^2^College of Agriculture and Life Sciences, Cornell University, Ithaca, NY, United States

**Keywords:** cassava, resistance, breeder's, pathologist's, cassava brown streak disease

## Abstract

Cassava production and productivity in Eastern, Central, and Southern Africa are ravaged by cassava brown streak disease (CBSD), causing yield losses of up to 100% when susceptible varieties are grown. Efforts to develop CBSD-resistant clones are underway. However, the methods for screening CBSD resistance currently vary between breeders and pathologists, with the limited empirical data to support their choices. In this study, we used the empirical CBSD foliar and root necrosis data from two breeding populations, termed cycle zero (C_0_) and cycle one (C_1_), to assess and compare the effectiveness of the CBSD screening methods of breeders vs. pathologists. On the one hand, the estimates of broad-sense heritability (*H*^2^) for the CBSD root necrosis assessment of breeder ranged from 0.15 to 0.87, while for the assessment method of pathologists, *H*^2^ varied from 0.00 to 0.71 in C_0_ clones. On the other hand, the marker-based heritability estimates (*h*^2^) for C_0_ ranged from 0.00 to 0.70 for the assessment method of breeders and from 0.00 to 0.63 for the assessment method of pathologists. For cycle one (C_1_) population, where both foliar and root necrosis data were analyzed for clones assessed at clonal evaluation trials (CETs) and advanced yield trials (AYTs), *H*^2^ varied from 0.10 to 0.59 for the assessment method of breeders, while the *H*^2^ values ranged from 0.09 to 0.35 for the CBSD computation method of pathologists. In general, higher correlations were recorded for foliar severity from the assessment method of breeders (*r* = 0.4, *p* ≤ 0.01 for CBSD3s and *r* = 0.37, *p* ≤ 0.01 for CBSD6s) in C_1_ clones evaluated at both clonal and advanced breeding stages than from the approach of pathologists. Ranking of top 10 C_1_ clones by their indexed best linear unbiased predictors (BLUPs) for CBSD foliar and root necrosis showed four overlapping clones between clonal and advanced selection stages for the method of breeders; meanwhile, only a clone featured in both clonal and advanced selection stages from the CBSD assessment method of pathologists. Overall, the CBSD assessment method of breeders was more effective than the assessment method of pathologists, and thus, it justifies its continued use in CBSD resistance breeding.

## Introduction

The human population in the next 30 years is projected to increase by 25%, from the current world population of ~7.5 billion to 10 billion people. The highest rate of this growth is expected to arise from sub-Saharan Africa (SSA; Hickey et al., 2017). Consequently, there is an urgent need to match this rapid growth in the human population with a concomitant increase in food production. Cassava (*Manihot esculenta* Crantz), a climate-resilient food staple in SSA, is a suitable crop to meet the projected calorie demand since more than half of the global production is in Africa (FAOSTAT, 2019).

Unfortunately, the average on-farm yield of cassava in Africa is low, stagnating at 12 tons/ha compared with 20 tons/ha estimated for Asian and Latin American countries (Malik et al., 2020). The biotic factors, such as cassava brown streak disease (CBSD), cassava mosaic disease (CMD), cassava bacterial blight, and whitefly vector, are the key obstacles to optimal cassava production and productivity in Africa (Maruthi et al., 2005; Mware et al., 2009; Patil and Fauquet, 2009; Patil et al., 2015). In the case of East Africa, the CBSD is currently the most devastating constraint for cassava production, causing yield losses of up to 100% in highly susceptible varieties (Alicai et al., 2007; Legg et al., 2011; Hillocks and Maruthi, 2015). Typical cassava plants infected with CBSD present characteristic yellowing along the veins, compromising the photosynthetic capacity of leaves, brown streaks on stems, and corky necrosis in the edible root parenchyma, and rendering the roots unusable for food or feed (Hillocks, 2004; Patil et al., 2015; Hillocks et al., 2016).

The severity and incidence of foliar and root CBSD symptoms form the basis of CBSD resistance screening. Currently, a scale of 1–5 is used to independently assess CBSD severity on foliar and roots; these assessments are commonly performed at 3 (CBSD3s) and 6 (CBSD6s) months for foliar and at 12 (CBSDRs) months at harvest for root necrosis (Hillocks, 2004; Kaweesi et al., 2014; Okul et al., 2018). The scores for the foliar severity assessment are as follows: 1 = no symptom, 2 = slight foliar chlorotic leaf mottle with no stem lesions, 3 = foliar chlorotic leaf mottle and blotches with mild stem lesions, 4 = foliar chlorotic leaf mottle and blotches with well-pronounced stem lesions, but no dieback, and 5 = defoliation with stem lesions and dieback. The scores for the root necrosis assessment are as follows: 1 = no necrosis, 2 = mild necrotic lesions (1–10%), 3 = pronounced necrotic lesions (11–25%), 4 = severe necrotic lesion (26–50%), and 5 = very severe necrotic lesions (>50%).

Although the CBSD symptom expressions are common to both breeders and pathologists, there is an apparent discrepancy in the data processing for decision support. For example, pathologists compute plot scores by averaging all severity scores ≥2, i.e., they exclude the CBSD severity scores of 1 when deriving plot mean for foliar and root symptoms (Ogwok et al., 2012; Odipio et al., 2014; Wagaba et al., 2017). On the other hand, breeders compute the averages of CBSD foliar and root severity using all the recorded observations, i.e., they do not exclude the CBSD scores of 1 (Kawuki et al., 2016, 2019; Okul et al., 2018). Essentially, the average values obtained from the CBSD assessments of pathologists or breeders are the different traits used for decision support.

In our efforts to optimize the cassava breeding operations tailored toward increased genetic gains, there is a need to assess the precision and relationship between the CBSD assessment methods. A key metric used to assess trait reliability is heritability, which measures the ratio of genetic variance to phenotypic variance (broad-sense heritability) or the ratio of additive genetic variance to phenotypic variance (narrow-sense heritability) (Bernardo, 2003). Accordingly, the data sets presented in this study aimed at answering the following research questions: (a) What proportion of total genetic and additive genetic variances are captured by the CBSD assessment methods of breeders and pathologists? and (b) To what extent do the CBSD assessment methods of breeders and pathologists select and advance the same clones?

## Materials and Methods

### Test Clones and CBSD Field Evaluations

The clones used in this study comprised genomic selection cycle zero (C_0_) and cycle one (C_1_) populations developed by the cassava breeding program of National Crops Resources Research Institute (NaCRRI). The data for C_0_ clones presented in this study were collected from clonal evaluation trials (CETs), while C_1_ clones were evaluated in both CETs and advanced yield trials (AYTs). The first set of CETs from C_0_, herein referred to as CETs-1, were evaluated at seven sites during first (April–May) and second (September–October) planting seasons in 2015. The first and second plantings generally depict the onset of rains. In Uganda, our first and second rains typically appear in February–March and September–October, respectively. The trial sites represent some of the key cassava production and consumption zones in Uganda. In these multilocational trials, a total of 155 C_0_ clones from a genomic selection training population of 427 genotypes were evaluated (Ozimati et al., 2018). Each trial was established in an augmented design with five checks (i.e., UG110008, UG110014, UG110015, UG110016, and UG110017) and replicated —five to six times in single-row plots of 10 plants spaced at 1 × 1 m between and within rows.

On the other hand, the C_1_ population presented in this study was generated from crosses made among 100 progenitors, a subset of the 155 C_0_ clones. In 2015–2016, we started with a seedling evaluation of ~5,000 genotypes for C_1_, from which 735 clones were evaluated in CET (2016–2017), herein referred to as CETs-2 at two locations (i.e., Namulonge and Serere). The CETs-2 were also planted in an augmented design with three checks, namely, UG110015, UG110017, and UG110134 in single-row plots of 10 plants spaced at 1 × 1 m between and within rows. During harvest in August 2017, a subset of 50 C_1_ clones were selected, based on the yield performance and response to CBSD as well as CMD from the CETs-2, and established in AYTs at three locations (i.e., Arua, Serere, and Namulonge). At each location, the trials were established in randomized complete block design, with a plot size of 6 × 6 m, replicated twice. For all trials, the plots were separated by 2-m alleys.

Since the plant-based foliar CBSD data collected at 3 (CBSD3s) and 6 (CBSD6s) months after planting (MAP) were only available for C_1_ clones assessed at CETs-2 and AYTs, we derived the mean foliar CBSD values for the assessment methods of breeders and pathologists for this population. To compute the plot means for foliar CBSD severity for the two disease assessment methods, plant-based diseases scored on a scale of 1–5 were used. In this case, score 1 = no foliar symptom expressions, 2 = mild symptoms (1–10%), 3 = pronounced chlorotic mottle and mild stem lesions (11–25%), 4 = foliar chlorotic leaf mottle and blotches with pronounced stem lesions (26–50%), and 5 = defoliation with stem lesions and dieback (>50%) (Hillocks and Thresh, 2000).

At harvest, which coincided with 12 MAP for both C_0_ and C_1_ populations, all plants per plot were uprooted, and roots were also assessed individually for CBSD necrosis using the scale of 1–5, where 1 = no necrosis, 2 = mild necrotic lesions (1–10%), 3 = pronounced necrotic lesions (11–25%), 4 = severe necrotic lesions (26–50%) with mild root constrictions, and 5 = very severe necrotic lesions (>50%) with severe root constrictions (Hillocks and Thresh, 2000; Kaweesi et al., 2014). We further processed the root necrosis data to match the mean CBSD severity computation methods of breeders and plant pathologists, i.e., all root severity scores were averaged for the assessment method of breeders, while only the root severity scores ≥2 were averaged for the CBSD assessment method of pathologists.

### Genotyping of the Clones

DNA was extracted from ~100 mg of fresh young leaves from each of the 155 C_0_ clones. DNA extractions were performed using QIAGEN DNeasy, Texas, USA extraction kits and quantified using Picogreen® to ensure that the required concentrations for sequencing were obtained. Consequently, DNA samples were genotyped using the genotyping-by-sequencing method as described by Elshire et al. (2011). Removing the single nucleotide polymorphic (SNP) markers by filtering and imputation methods has been described in an earlier study (Hamblin and Rabbi, 2014; Wolfe et al., 2016, 2017). Ultimately, we had a total of 25,383 SNP markers, which were filtered at minor allele frequency (MAF) ≥0.01 for the estimation of SNP-based heritability for each of the C_0_ clones.

### Statistical Analyses

To estimate the broad-sense heritability for each CBSD assessment method, i.e., breeders vs. pathologists for C_0_ clones, we fitted the linear mixed model for each trial using the *lme4* package for the R statistical computing software (R Development Core Team, 2008) as follows:

$$\begin{array}{l} {\text{y}}_{\text{ijk}}=\text{ μ}+\;{{\text{c}}_{\text{i}}+\text{b}}_{\text{j}}+\;{\text{e}}_{\text{ijk}}\text{ Model }1 \end{array}$$

where **y**_**ijk**_ was the response of **i**th clone from **j**th block in the **k**th plot, **μ** represented the fixed trial mean, **b** and **c** represented a vector of random block and clone effects, respectively, and **e** was the random residual term. The variance components to compute the broad-sense heritability (*H*^2^) were extracted from the model described earlier. The plot-based broad-sense heritability estimates for root necrosis for the two CBSD assessment methods across 14 CETs-1 (i.e., location–season combinations) were then computed as follows:

$$H^{2}=\frac{\sigma_{\text{c}}^{2}}{\left(\sigma_{\text{c}}^{2}+\sigma_{\text{b}}^{2}+\sigma_{\text{e}}^{2}\right)}$$

where $\sigma_{\text{c}}^{2}$ was the clone variance, $\sigma_{\text{b}}^{2}$ was the variance due to blocks, and $\sigma_{\text{e}}^{2}$ was the model residual variance.

To obtain the genomic estimated breeding values and the additive genetic variance for the two methods from CETs-1, we fitted a single-step genomic best linear unbiased predictor (G-BLUP) model as follows:

$$\begin{array}{l} {\text{y}}_{\text{ijk}}=\text{ μ}+\;{\text{w}}_{\text{i}}+\;{\text{g}}_{\text{j}}+\;{\text{e}}_{\text{ijk}}\text{ Model }2 \end{array}$$

where **y**_ijk_ was the response of **j**th genotype in the **i**th block recorded for **k**th plot, **μ** and **w** were the fixed grand mean and block effects, respectively, **g**_j_ represented the random genotype effect, assuming **g**_j_ **~** **N(0, G**$\sigma_{\text{g}}^{2}$) with $\sigma_{\text{g}}^{2}$ representing the variance due to genotypic effects while **G** represented the covariance structure among clones based on the marker data, and **e** was the random model residual effect, assumed to be normally distributed as $\varepsilon_{\text{ijk}}^{\text{IID}}$ **~** **N (0**, $\sigma_{\text{e}}^{2}$**)** with $\sigma_{\text{e}}^{2}$ as the residual variance. We extracted the variance components from the G-BLUP model and estimated the narrow-sense heritability (*h*^2^ SNP-heritability) using the formula as follows:

$${\text{h}}^{2}=\frac{\sigma_{\text{g}}^{2}}{\left(\sigma_{\text{g}}^{2}+\sigma_{\text{e}}^{2}\right)}$$

where $\sigma_{\text{g}}^{2}$ was the additive genetic variance and $\sigma_{\text{e}}^{2}$ was the model residual variance.

Furthermore, we examined how many top 10 ranked clones at CETs-2 were featured among the best 10 clones at AYTs for the two CBSD assessment methods from C_1_ population. To do this, the data sets from each of the two trial stages (i.e., CETs-2 and AYTs) were combined across sites, followed by fitting a multilocational linear mixed model as described below for each trial stage. For the CETs-2, we fitted a multilocational model described as follows:

$$\begin{array}{l} {\text{y}}_{\text{ijkl}}=\text{ μ}+\;{\text{l}}_{\text{i}}+\;{\text{g}}_{\text{j}}+{\text{b}/\text{l}}_{\text{k}\left(\text{i}\right)}+{\text{gl}}_{\text{ij}}+\;{\text{ε}}_{\text{ijkl}}\text{ Model }3 \end{array}$$

where the grand mean **μ** and the main effect of the **i**th environment **(l)** were considered fixed, while the **j**th genotype **(g)**, the **k**th block **(b)** nested within the **i**th environment **(l)**, the interaction of the **j**th genotype **(g)** by **i**th environment **(gl)**, and the residual term **(ε)** were considered random. The variance components were extracted for the estimation of broad-sense heritability, using the formula described above for CETs-1.

Similarly, we fitted a multilocational linear mixed model for C_1_ AYTs, where the grand mean and location were considered fixed, while clones, replicates nested within trial, genotype-by-environment interactions, and residual terms were considered random. Accordingly, the variance components were extracted to compute the plot-based broad-sense heritability estimates for foliar and root necrosis for the two CBSD assessment methods.

The raw phenotypic means and BLUP values for foliar CBSD severity as well as root necrosis of C_1_ clones were extracted for both CETs-2 and AYTs from the models fitted and used to compute Pearson's correlation coefficients for 50 C_1_ clones that featured in both CETs-2 and AYTs for each of the CBSD assessment methods. Furthermore, we computed selection index (SI) from BLUPs and raw phenotypic means of the three traits across sites, with the traits having equal economic weights as follows:

$$\begin{array}{l} \text{SI}=-1\left(\text{CBSD}3\text{s}\right)+-1\left(\text{CBSD}6\text{s}\right)+-1\left(\text{CBSDRs}\right) \end{array}$$

where CBSD3s, CBSD6s, and CBSDRs were the CBSD severities assessed at 3, 6, and 12 MAP, respectively.

Finally, we used the indexed BLUP values of the three traits for the 50 clones that appeared at both CETs-2 and AYTs for ranking the top 10 clones at each trial stage. The purpose of ranking was to compare the number of 10 top clones that overlapped at CETs-2 and AYTs for each of the CBSD averaging methods.

## Results

### Broad-Sense and SNP-Heritability Estimates

The broad-sense heritability (*H*^2^) estimates for the CBSD root severity assessment method of breeders ranged from 0.15 in Arua 2015A trial to 0.87 in Namulonge 2015A trial (Table 1). On the other hand, *H*^2^ estimates for the assessment method of pathologists ranged from 0.00 in Arua 2015A trial to 0.71 in Namulonge 2015A and B trials (Table 1). Meanwhile, the narrow-sense heritability (*h*^2^) estimates, also referred to as SNP-based heritability, for the assessment method of breeders ranged from 0.00 in Arua 2015A trial to 0.72 in Namulonge 2015A trial (Table 1). Similarly, *h*^2^ for the assessment method of pathologists varied from 0.00 in Arua 2015A trial to 0.63 in Serere 2015A trial. Overall, the average broad-sense and narrow-sense heritability estimates across trials were higher for the CBSD assessment method of breeders (*H*^2^ = 0.56 and *h*^2^ = 0.36) than for the CBSD assessment approach of pathologists (*H*^2^ = 0.49 and *h*^2^ = 0.25) (Table 1).

**Table 1:** Broad and narrow-sense heritability estimates associated with breeder's and pathologist's CBSD root severity assessment methods.

**Trial location**	**Seasons**	**C_**0**_ clones**	**Broad-sense heritability**		**Narrow-sense heritability**	
			**Breeder's**	**Pathologist's**	**Breeder's**	**Pathologist's**
Mityana	2015A	115	0.39	0.51	0.10	0.04
Mityana	2015B	105	0.62	0.64	0.49	0.22
Arua	2015A	149	0.15	0.00	0.00	0.00
Arua	2015B	111	0.64	0.47	0.39	0.34
Kasese	2015A	116	0.26	0.06	0.21	0.06
Kasese	2015B	138	0.54	0.57	0.51	0.34
Kigumba	2015A	147	0.49	0.61	0.25	0.09
Kigumba	2015B	116	0.56	0.54	0.13	0.05
Namulonge	2015A	150	0.87	0.71	0.72	0.31
Namulonge	2015B	113	0.79	0.71	0.55	0.45
Serere	2015A	123	0.68	0.64	0.64	0.63
Serere	2015B	112	0.71	0.58	0.70	0.56
Lira	2015A	149	0.47	0.44	0.25	0.22
Lira	2015B	108	0.67	0.48	0.54	0.27
Mean Heritability			0.56	0.49	0.39	0.25

*2015A and 2015B, refers to the first (April-May) and second (Aug-Sept) planting seasons*.

**Figure 1:**
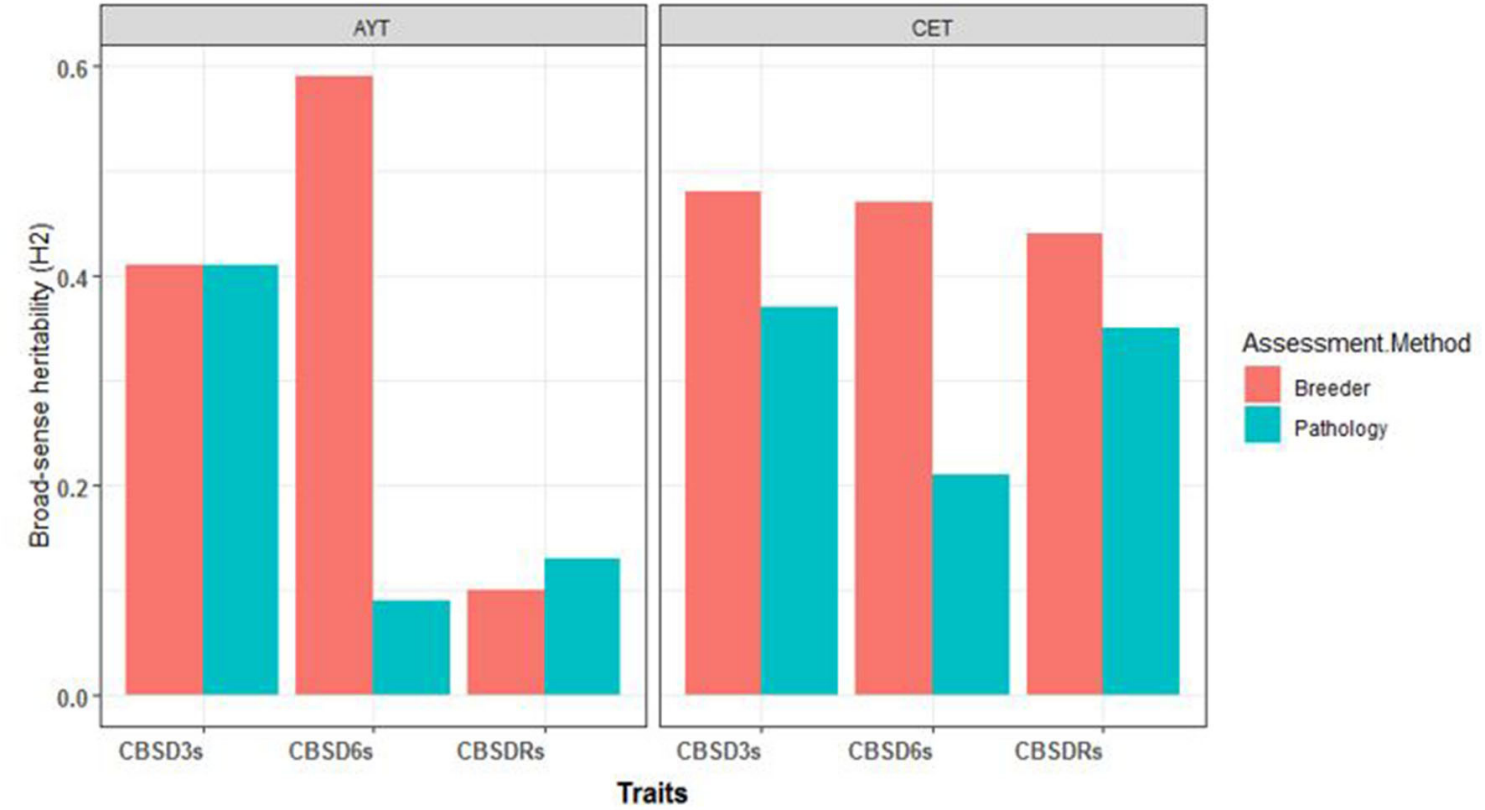
The broad-sense heritability estimates (H^2^) for the three disease traits (cassava brown streak disease severity assessed at 3-months after planting [CBSD3s], cassava brown streak disease severity assessed at 6-months after planting [CBSD6s], cassava brown streak disease root severity assessed at 12-months after planting [CBSDRs]) for clonal evaluation trials (CETs-2) and advanced yield trials (AYTs) for the two mean CBSD computation methods.

For C_1_ population, the broad-sense heritability estimates for foliar and root necrosis from both CETs-2 and AYTs are presented in Figure 1. We also observed higher *H*^2^ values for the CBSD assessment method of breeders compared with the CBSD assessment method of pathologists for both CET and AYT evaluation stages. For example, at CET, *H*^2^ at 3 months was 0.48 for the method of breeders and 0.38 for the method of pathologists. At 6 months, *H*^2^ was 0.47 for the method of breeders and 0.21 for the computation of pathologists. Based on the root necrosis data at harvest, the broad-sense heritability values were 0.44 and 0.35 for the methods of breeders and pathologists, respectively. Similarly, the higher broad-sense heritability estimates of 0.42 and 0.56 were recorded for the combined data from AYTs for the method of breeders compared with the estimates of 0.41 and 0.09 recorded for the computations of pathologists for CBSD3s and CBSD6s, respectively (Figure 1).

### Relationship Between BLUP Values of the 50 Clones Evaluated at CETs-2 and AYTs for Mean CBSD Assessment Methods

In general, we recorded higher Pearson's correlation coefficients from the foliar CBSD assessment method of breeders than the approach of pathologists, using both BLUP estimates and raw phenotypic means across locations (Table 2). On the one hand, the highest correlation coefficient value (*r* = 0.40, *p* ≤ 0.01) was observed for CBSD3s from the assessment method of breeders. On the other hand, low and statistically nonsignificant correlation coefficients were recorded for root necrosis and indexed trait values for both the CBSD assessment methods (Table 2). The correlation values for root necrosis and indexed trait values varied from 0.02 to 0.21. Overall, for all three disease traits and their indexed values, the CBSD computation method of breeders had higher correlation coefficients than the approach of the CBSD assessment of pathologists (Table 2).

**Table 2:** Pearson correlation coefficients of the 50 clones evaluated at CETs-2 and AYTs.

**BLUPs**			**Raw phenotype**	
**Traits**	**Breeder's**	**Pathologist's**	**Breeder's**	**Pathologist's**
CBSD3s	0.40**	0.29**	0.37**	0.19^ns^
CBSD6s	0.37**	0.20^ns^	0.36**	0.26*
CBSDRs	0.02^ns^	0.03^ns^	0.05^ns^	0.11^ns^
S.I	0.20^ns^	0.06^ns^	0.21^ns^	0.09^ns^

*, **Significant correlation at p ≤ 0.05 and 0.01, respectively.

*ns, non-significant correlations coefficients; CBSD3s, cassava brown streak disease severity scored 3 months after planting; CBSD6s, cassava brown streak disease severity scored 6 months after planting; CBSDRs, cassava brown streak disease root severity scored at 12-months harvest; S.I, selection index values for the three cassava brown streak traits; BLUPs, best linear unbiased predictors for clones*.

### Ranking of 50 Clones in CETs-2 and AYTs Using Indexed BLUPs Values for the Two CBSD Averaging Methods

We ranked the 50 clones from C_1_, CETs-2, and AYTs by their indexed BLUP values of CBSD3s, CBSD6s, and CBSDRs for the two CBSD assessment methods (Table 3). Based on ranking of the top 10 clones, four clones (i.e., UG15F190P001, UG15F170P507, UG15F079P011, and UG15F176P502) evaluated in CETs-2 and AYTs overlapped among the top 10 ranked clones for the mean CBSD assessment method of breeders, whereas only one clone (UG15F190P001), overlapped between CETs-2 and AYTs evaluated among the top 10 ranked clones (Table 3).

**Table 3:** Ranking of top 10 C_1_ clones by their indexed BLUPs values for the two CBSDs.

**Breeder's**		**Pathologist's**	
**CETs-2**	**AYTs**	**CETs-2**	**AYTs**
UG15F190P001^*^	UG15F190P001	UG15F262P513	UG15F265P001
UG15F262P513	UG15F079P011	UG15F190P001^*^	UG15F312P003
UG15F170P507^*^	UG15F140P003	UG15F017P003	UG15F190P001
UG15F176P004	UG15F196P004	UG15F177P016	UG15F249P007
UG15F201P517	UG15F176P502	UG15F170P507	UG15F047P010
UG15F079P011^*^	UG15F177P016	UG15F306P028	UG15F044P009
UG15F176P502^*^	UG15F044P009	UG15F176P004	UG15F169P507
UG15F017P003	UG15F170P507	UG15F222P038	UG15F158P005
UG15F209P001	UG15F222P038	UG15F361P510	UG15F140P001
UG15F302P513	UG15F312P003	UG15F154P005	UG15F196P004

*CETs-2, clonal evaluation trial (C1); AYTs, advanced yield trials*.

* *Overlapping clones at CET and AYT*.

## Discussion

On recognizing the CBSD epidemic in Uganda in the early 2000s, concerted research efforts were initiated to understand the diversity of viruses causing CBSD (Mbanzibwa et al., 2011; Alicai et al., 2016; Ateka et al., 2017; Mbewe et al., 2017), their transmission by the whitefly vector, *Bemisia tabaci* (Maruthi et al., 2005; Omongo et al., 2012; Mugerwa et al., 2018; Ally et al., 2019), and sourcing for resistant genetic materials for breeding (Kanju et al., 2007; Kawuki et al., 2016). More recently, transgenic approaches have also been explored to combat CBSD, but with no officially released genetically transformed plant under cultivation in Uganda to date (Patil et al., 2011; Yadav et al., 2011; Wagaba et al., 2017). Collectively, these research interventions have contributed to our increased understanding and management of CBSD.

A discrepancy remains in the methodologies of CBSD resistance screening, which continues to be refined (Kawuki et al., 2019). In general, in screening for CBSD resistance, plant pathologists assess clone performance based on average foliar infected plants and/or roots, i.e., exclude scores of 1 (Ogwok et al., 2012; Odipio et al., 2014). On the other hand, breeders assess clone performance based on average foliar infected plants and/or roots without excluding the severity scores of 1, i.e., no data are excluded (Kanju et al., 2007; Okul et al., 2018; Kawuki et al., 2019; Ozimati et al., 2019). Certainly, the methods have varying sampling sizes, hence introducing sampling errors or biases. This study aimed at comparing the two CBSD severity assessment methods based on the heritability estimates and the relative ranking of clones at different trial stages.

### Heritability Estimates of CBSD Foliar and Root Necrosis for the Two Assessment Methods

According to Bernardo (2003), the broad- and narrow-sense heritability estimates are critical for selection decisions. The comparison of heritability estimates across CETs-1 revealed higher heritability estimates for the method of breeders for CBSD root severity assessment than that for the method of pathologists, with the highest plot-based broad-sense (*H*^2^ = 0.87) and narrow-sense (*h*^2^ = 0.72) heritability estimates recorded for Namulonge trial in 2015A. In a recent study by Kawuki et al. (2019), a minimum number of 30 roots per plot were recommended to obtain the meaningful assessment of CBSD root necrosis. A notable difference between the CBSD assessment methods of breeders and pathologists is that the former uses sample sizes larger (i.e., includes all roots to obtain plot mean) than the latter (i.e., excludes roots with a severity score of 1). Averaging all root scores per plot possibly explains the higher precision and heritability estimates observed for the CBSD assessment of breeders compared with that for the approach of pathologists with the exclusion of roots scores of 1 (i.e., no necrosis). In the same study by Kawuki et al. (2019), the lowest standard error from five CBSD root necrosis assessment methods were associated with trials at Namulonge, supporting early studies qualifying Namulonge as a hot spot for CBSD screening (Kaweesi et al., 2014; Okul et al., 2018). It is not surprising that Namulonge presented the highest heritability estimates in this study, supporting it as a hot spot for CBSD screening. Efforts are currently in place to improve the CBSD phenotyping at the hot spot in Namulonge by the use of imaging technology, which is considered a robust and less subjective screening method. As stated by Bernardo (2003), heritability is an important function in the genetic study of metric character, because it reflects the predictive accuracy and reliability of the phenotypic values. Thus, the highest heritability estimates (i.e., broad sense and narrow sense) for both foliar and CBSD root necrosis recorded from the computation of breeders support the use of this method for efficient selection of CBSD-resistant clones.

### Comparing Pearson's Correlation Coefficients for BLUP Estimates of Clone in CETs-2 and AYTs for the Two CBSD Assessments Methods

The best linear unbiased predictor (BLUP) pioneered by C.R. Henderson (Piepho et al., 2008) as a procedure for genetic estimation was first used for practical dairy breeding. The BLUP procedure allows for a more accurate estimation of genetic merit of traits in the unbalanced data while accounting for the differences in the amount of data available for each genotype (Bernardo, 2003). In general, the correlation coefficients of BLUP values for CBSD traits of clones that were filtered from CETs-2 (C_1_) to AYTs (C_1_) were low to moderate (*r* = 0.02–0.40). However, these correlation coefficients were higher and significant (*p* ≤ 0.01) for the mean foliar CBSD computation of breeders than the method of pathologists for clones that made it from CETs-2 to AYTs. Ozimati et al. (2019) previously reported a high genetic correlation of 0.70 for root necrosis between measurements at seedling vs. at clonal evaluations. In this study, the low correlation observed between BLUPs values at CETs-2 and AYTs for root necrosis could be due to degeneration. Recycling the clones for more than three planting seasons has been reported to cause resistance degeneration due to the buildup of the virus population (Shirima et al., 2017). In fact, to date, no clones have been reported to be immune in the conventional breeding pipeline, except for the recent sources of immunity reported from Latin American germplasm (Sheat et al., 2019). One approach of selecting and advancing clones in face of degeneration due to the virus buildup would be to complement the symptom-based screening with the measurements of virus titer, especially when advancing clones from the mid-to-late stages of selection, i.e., from CET stage onward. However, the high cost per assay is a major limitation to the use of quantitative PCR (q-PCR) for virus screening of a large number of clones, as at CET (i.e., over 600 genotypes; Ogwok et al., 2012; Kaweesi et al., 2014; Okul et al., 2018). Through international collaboration with Plant Virus Department, Leibniz Institute DSMZ-German Collection of Microorganism and Cell Culture, Braunschweig, Germany, a cheap and rapid assay is being developed to enable the screening of large entries. Nonetheless, the higher correlation coefficients observed between the BLUP values of clones in CETs-2 and AYTs for mean CBSD computation of breeders than for the approach of pathologists support the use of the assessment methods of breeders for a more effective selection of resistant clones.

### Ranking of Clones by Their Indexed BLUPs for the Two CBSD Averaging Methods

In a recent study by Kawuki et al. (2019), to evaluate the alternative methods for assessing CBSD root necrosis, 256 clones were ranked using their BLUPs for five CBSD assessment methods. The comparison of the top 15 resistant clones ranked across the CBSD assessment methods showed one overlapping clone for all the five CBSD root necrosis assessment methods (Kawuki et al., 2019). In this study, ranking of the top 10 resistant clones from CETs-2 and AYTs revealed four clones featuring at both evaluation stages for the CBSD assessment method of breeders compared with only a single clone that overlapped for the approach of pathologists. Four clones overlapping at CETs-2 and AYTs for breeders mean CBSD computation relative to a single clone for pathologists assessment method, further supports the use of breeders-derived phenotypes to guide selection decisions.

## Conclusion

This study provides insights into CBSD necrosis assessment as performed by the methods of breeders and pathologists that remarkably differ in how the mean severities are computed. Based on the heritability estimates and the number of clones that were filtered, it was evident that computing mean CBSD for the entire number of roots from a plot was more reliable compared with cases where roots with severity scores of 1 were excluded.

## Data Availability Statement

The original contributions presented in the study are included in the article/Supplementary Material, further inquiries can be directed to the corresponding author/s.

## Author Contributions

AO conceived the original manuscript idea, analyzed the data, and wrote the manuscript. RK, TA, WE, J-LJ, and CE reviewed the manuscript. All authors contributed to the article and approved the submitted version.

## Conflict of Interest

The authors declare that the research was conducted in the absence of any commercial or financial relationships that could be construed as a potential conflict of interest.
